# The role of rare compound heterozygous events in autism spectrum disorder

**DOI:** 10.1038/s41398-020-00866-7

**Published:** 2020-06-22

**Authors:** Bochao Danae Lin, Fabrice Colas, Isaac J. Nijman, Jelena Medic, William Brands, Jeremy R. Parr, Kristel R. van Eijk, Sabine M. Klauck, Andreas G. Chiocchetti, Christine M. Freitag, Elena Maestrini, Elena Bacchelli, Hilary Coon, Astrid Vicente, Guiomar Oliveira, Alistair T. Pagnamenta, Louise Gallagher, Sean Ennis, Richard Anney, Thomas Bourgeron, Jurjen J. Luykx, Jacob Vorstman

**Affiliations:** 1 Department of Psychiatry, Brain Center Rudolf Magnus, University Medical Center Utrecht, Utrecht University, Utrecht, The Netherlands; 2 Department of Preventive Medicine, Institute of Biomedical Informatics, Bioinformatics Center, School of Basic Medical Sciences, Henan University, Kaifeng, China; 3 Department of Translational Neuroscience, Brain Center Rudolf Magnus, University Medical Center Utrecht, Utrecht University, Utrecht, The Netherlands; 4 Department of Medical Informatics, University Medical Center Utrecht, Utrecht University, Utrecht, The Netherlands; 5 Institute of Neuroscience, Newcastle University, Newcastle, UK; 6 Division of Molecular Genome Analysis and Division of Cancer Genome Research, German Cancer Research Center (DKFZ), Heidelberg, Germany; 7 Department of Child and Adolescent Psychiatry, Psychosomatics and Psychotherapy, University Hospital Frankfurt, JW Goethe University Frankfurt, Frankfurt am Main, Germany; 8 Department of Pharmacy and Biotechnology, University of Bologna, Bologna, Italy; 9 Department of Psychiatry, University of Utah School of Medicine, Salt Lake City, UT USA; 10 Instituto Nacional de Saúde Doutor Ricardo Jorge, Avenida Padre Cruz, Lisboa, Portugal; 11 Centro Hospitalar de Coimbra, Coimbra, Portugal; 12 NIHR Oxford BRC, Wellcome Centre for Human Genetics, University of Oxford, Oxford, UK; 13 Neuropsychiatric Genetics Research Group, Department of Psychiatry, Trinity College Dublin, Trinity Centre for Health Sciences, Dublin, Ireland; 14 Academic Centre on Rare Diseases, School of Medicine and Medical Science, University College Dublin, Dublin, Ireland; 15 Medical Research Council Centre for Neuropsychiatric Genetics and Genomics, Division of Psychological Medicine and Clinical Neurosciences, School of Medicine, Cardiff University, Cardiff, UK; 16 Human Genetics and Cognitive Functions, Institut Pasteur, UMR3571 CNRS, Université de Paris, Paris, France; 17 GGNet Mental Health, Apeldoorn, The Netherlands; 18 Program in Genetics and Genome Biology, Research Institute, and Department of Psychiatry, The Hospital for Sick Children, Toronto, ON Canada; 19 Department of Psychiatry, University of Toronto, Toronto, ON Canada

**Keywords:** Autism spectrum disorders, Comparative genomics

## Abstract

The identification of genetic variants underlying autism spectrum disorders (ASDs) may contribute to a better understanding of their underlying biology. To examine the possible role of a specific type of compound heterozygosity in ASD, namely, the occurrence of a deletion together with a functional nucleotide variant on the remaining allele, we sequenced 550 genes in 149 individuals with ASD and their deletion-transmitting parents. This approach allowed us to identify additional sequence variants occurring in the remaining allele of the deletion. Our main goal was to compare the rate of sequence variants in remaining alleles of deleted regions between probands and the deletion-transmitting parents. We also examined the predicted functional effect of the identified variants using Combined Annotation-Dependent Depletion (CADD) scores. The single nucleotide variant-deletion co-occurrence was observed in 13.4% of probands, compared with 8.1% of parents. The cumulative burden of sequence variants (*n* = 68) in pooled proband sequences was higher than the burden in pooled sequences from the deletion-transmitting parents (*n* = 41, *X*^2^ = 6.69, *p* = 0.0097). After filtering for those variants predicted to be most deleterious, we observed 21 of such variants in probands versus 8 in their deletion-transmitting parents (*X*^2^ = 5.82, *p* = 0.016). Finally, cumulative CADD scores conferred by these variants were significantly higher in probands than in deletion-transmitting parents (burden test, *β* = 0.13; *p* = 1.0 × 10^−^^5^). Our findings suggest that the compound heterozygosity described in the current study may be one of several mechanisms explaining variable penetrance of CNVs with known pathogenicity for ASD.

## Introduction

Autism spectrum disorders (ASDs) are a group of neurodevelopmental disorders characterized by social and communicative deficits, a marked insistence on sameness and/or repetitive behaviors^1^. The estimated population prevalence of ASDs is ~1%^2^. It is well established that genetic factors contribute to the risk of ASDs^3^. The identification of the genetic risk variants associated with ASDs constitutes an appealing strategy to elucidate their underlying biology^4,5^. Genetic variants identified so far include single nucleotide variants (SNVs), as well as structural abnormalities in copy number (CNVs), leading to a loss or gain of up to several millions of base pairs. These variants can be inherited or can occur de novo, i.e., a novel change in the genetic code emerges in the child while not part of the DNA sequence of either parent.

Common variants occur frequently in the population (minor allele frequency (MAF) of 5% or more) and are associated with small risk increases^6,7^. However, current estimates of the cumulative effect of such common variants account for 12% of the variance in autism (SNP heritability (*h*^2^ = 0.118)^7,8^. There is also evidence for the role of rare variants in ASD; these are alleles that occur infrequently in the population (e.g., MAF < 1%) but may be associated with larger risk effects in the individual carrier. It is estimated that causative rare genetic variants, both de novo and inherited, can be identified in 10–30% of patients with ASD^9–11^.

**Fig. 1:**
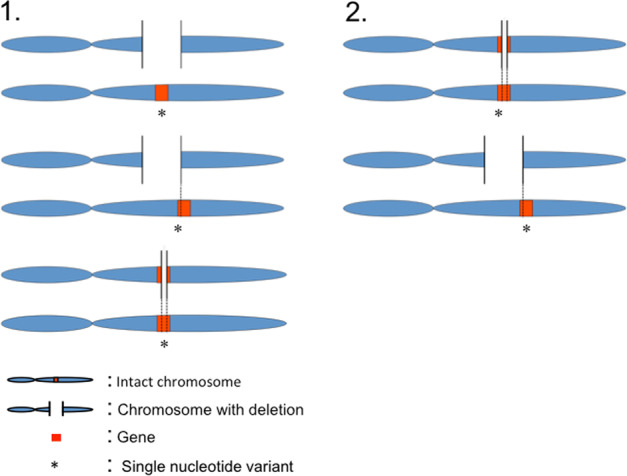
Different compound heterozygosity scenarios. Scenario 1: a gene is included, partly or entirely, in a deletion. A sequence variant occurs at the remaining allele of the gene, within the boundaries of the deleted region. Scenario 2: a gene is partly included in a deletion. A sequence variant occurs at the remaining allele of the gene, but outside the boundaries of the deleted region.

When a deletion affects a genomic region with optimally functioning genes on the remaining allele, the most likely effect of that deletion is a change in gene expression with potential to result in a phenotypic effect^12^. However, a pathogenic impact may be more likely if the performance of a gene on the remaining allele is also impacted by a functional variant (“compound heterozygosity”). The co-occurrence of impactful variation on both copies of a gene, a deletion on the one and a functional variant on the other allele, may thus be a relevant genetic mechanism in ASD (see Fig. 1). The psychiatric genetics literature provides precedents for this “double hit” mechanism, which can be considered as a specific type of compound heterozygosity: several case studies report the co-occurrence of an inherited deletion and a functional variant on the remaining allele in probands with autism^13–15^ and in schizophrenia^16,17^. Furthermore, the rate of a slightly different type of compound heterozygosity, i.e., two rare loss-of-function sequence variants co-occurring at the same locus, is found to be significantly increased in autism compared with controls^18,19^.

Here, we hypothesize that compound heterozygosity of a deletion and a functional sequence variant at the remaining allele occurs more often in patients with ASDs compared with their parents transmitting the deletions. We speculate that this compound heterozygosity mechanism may provide an explanation for the penetrance of the inherited CNVs identified in individuals with ASD, compared with unaffected parents. The current study aims to provide empirical evidence for the proposed compound heterozygosity mechanism as a relevant causative factor in a proportion of ASD cases.

## Material and methods

### Project overview

We selected proband–parent pairs and trios from an existing dataset (Autism Genome Project, AGP) of 2191 families for which previous studies had already provided data from genome-wide CNV screening^20^. In brief, diagnosis of ASD was based on standardized assessments and/or clinical evaluation, as described previously^20^. DNA samples were available from six European sites and one American site from the AGP. Ethical approval was obtained from all participating sites’ IRBs and all participants provided written informed consent. We collected DNA aliquots that remained after the major genetic analyses of the AGP had been performed^21–24^. We abided by the principles laid out in the Declaration of Helsinki.

**Fig. 2:**
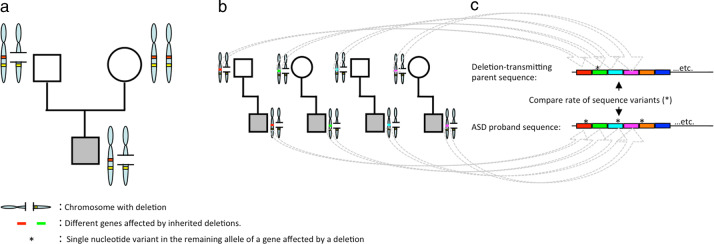
Schematic overview of the study. **a** Identification of inherited deletions in probands. In this example, the proband inherited a deletion from the father. The deletion involves one gene (red). We prioritized inherited deletions that involved one or more genes with probable relevance to the brain. **b** Targeted sequencing of deleted gene(s) in each proband and his/her parent(s) who transmitted the deletion. We analyzed 102 proband–parent pairs and 47 proband–parent trios. (in this figure, only proband–parent pairs are shown). **c** Comparing the rate of sequence variants (*) in the pooled set of sequenced genes between probands and their deletion-transmitting parents. For our analyses, for each of the 149 families we only queried the sequence of gene(s) affected by inherited deletion(s) in that specific family.

From the available AGP dataset we prioritized those probands who had inherited at least one deletion from a parent. We prioritized inherited deletions that involved one or more genes with probable relevance to the brain. We annotated genes as brain relevant on the basis of concordance between three different data categories: (1) sequence tags expressed in the brain (ESTs)^25^; (2) results from a large gene expression analysis^26^; and (3) biological functions inferred by matching a vocabulary of brain-related terms against gene ontologies from the AmiGO database^27^ (see Supplementary methods). After prioritization of subjects (see below), we investigated in our selected study population the rate of additional sequence variants in those genes affected by inherited deletions. We used targeted genomic enrichment followed by next-generation sequencing^28^ to identify the co-occurrences of inherited deletions with a functional sequence variant in the remaining allele in our entire sample of pedigrees. In essence, we examined the rate of these compound heterozygous events by comparing the sum of sequence variants in all deleted gene regions in probands to the sum of sequence variants identified in the same deleted gene regions in the parent who transmitted the deletion to each proband (Figs. 1 and 2). In addition, we investigated whether the cumulative predicted functional impact, as expressed by the Combined Annotation-Dependent Depletion v1.4 (CADD)^29^ scores (see below) of the genetic variants is different in probands compared with deletion-transmitting parents.

### DNA sample collection and subject prioritization steps

We considered families from the seven sites that participate in the AGP, i.e., France, Germany, United Kingdom (International Molecular Genetic Study of Autism families) England, Ireland, Italy, Portugal, and the United States. There were *N* = 2191 families (mostly trios) for a total of 6986 samples. We prioritized CNV calls based on the following criteria: (1) called by two or more algorithms (QuantiSNP^30^, PennCNV^31^, and iPattern^32^); (2) <10% frequency in the AGP dataset to exclude common CNVs that are likely to be benign; and (3) length >5 kb to ensure adequate reliability of CNV detection algorithms^33^.

Furthermore, we attempted to enrich the sample for families with a theoretically higher likelihood of a compound heterozygous event. To that end, first, we excluded families with more than one affected proband, given that the likelihood of the same compound heterozygous event in more than one proband in a multiplex family is <0.25, assuming that in a proportion of cases the origin of a functional sequence variant in the remaining allele is de novo. Second, under the assumption that homozygous deletions affecting brain-expressed genes are likely pathogenic, we excluded probands with homozygous deletions. Third, we prioritized those probands with at least one deletion involving one or more genes relevant to the brain (defined hereafter). Finally, genetic variants, even those considered highly pathogenic, are often not completely penetrant^34^, suggesting that additional genetic variants in the genome may contribute to phenotypic expression. Therefore, rather than categorically excluding certain families based on a likely pathogenic variant, we chose a prioritization strategy. Hence, we prioritized probands with the smallest numbers of de novo CNVs (deletions and duplications) as de novo CNVs are more likely causative, thereby reducing the likelihood of a causative compound heterozygous event. Finally, we prioritized probands with the largest number of inherited CNVs, in particular those involving brain-relevant genes, while attributing a double weight to deletions compared with duplications:

$$\begin{array}{l} R_{\rm{i}} = 2 \times \displaystyle\left( {R_{{\rm{N}}_{\rm{i}}^{{\mathrm{del}}}} + R_{{\rm{N}}_{\rm{i}}^{{\mathrm{brain}}\;{\mathrm{del}}}} + R_{{\rm{R}}_{{\mathrm{inherit}},{\rm{i}}}^{{\mathrm{del}}}}}\right)\\+\, 1 \times \left({R_{{\rm{N}}_{\rm{i}}^{{\mathrm{dup}}}} + R_{{\rm{N}}_{\rm{i}}^{{\mathrm{brain}}\;{\mathrm{dup}}}} + R_{{\rm{R}}_{{\mathrm{inherit}},{\rm{i}}}^{{\mathrm{dup}}}}}\right)\end{array}.$$

Applying these criteria to the AGP families, we retrieved DNA samples from the participating sites of 254 families.

### Targeted genomic enrichment and sequencing

We custom-designed a target sequence footprint, applying 60-mer tiling probes based on the selected genes for this study. Agilent SureSelect (Santa Clara) in solution capture assays were used for the enrichment procedure. The library preparation has been described in detail elsewhere^35^. Briefly, DNA samples were sheared into 100–120 nucleotide fragments, followed by ligation of double-stranded short adapters and, subsequently, ligation-mediated polymerase chain reaction (PCR) amplification. The pooled library fragments were then hybridized to the Agilent capture assays and underwent post enrichment PCR before sequencing.

We performed sequencing of enriched barcoded samples on a SOLiD 5500XL sequencer (Applied Biosystems) with V3 chemistry according to the manufacturer instructions to produce 50 bp sequencing reads. Reads were mapped onto the human genome (GRCh37), using BWA^36^ as default settings with the following parameters (-c -l 25 -k 2 -n 10).

### Variant calling and quality control

A custom PERL pipeline (https://github.com/UMCUGenetics/SAP42) was developed to parse the BAM files and extract SNP genotypes with the following criteria: at least 10× coverage, sequencing quality Q >20, >15% non-reference alleles at variant sites (this is a cut-off criterion for individual sample positions), and support from >3 independent reads on both strands. A maximum number of five identical reads calling the same allele is set to suppress excessive co-linearity effects. The genetic variants calling was performed for each sample from BAM files and then merged.

The processed VCF file contained 357 individuals from 161 families, with a total of 50,729 SNVs (47 complete trios and 102 proband–parent pairs, as well as 12 singletons without sequence data from their transmitting parents; these 12 singletons were excluded from further analysis). Variants were annotated using SnpEff software, version 4.3 T^37^. All results of this study are reported in GRCh37/hg19 build. The CNV regions previously reported in this sample^20^ were reported in NCBI/hg18build. CNV coordinates were re-mapped to GRCh37/hg19 build using a publicly available LiftOver application (https://genome.ucsc.edu/cgi-bin/hgLiftOver).

The gene content of a CNV was defined as all genes located within the CNV region; an additional 500 kb fuzzy border was applied at both the 5′ and 3′ ends of the reported CNV. We extracted all SNVs located in the genes affected by inherited deletions; thus, in this study compound heterozygotes were defined as a second variant occurring in the gene and within the boundaries of the deletion region (Fig. 1, scenario 1). Alternatively, a genic sequence variant can be identified in a gene affected by a deletion, but outside breakpoints of the deletion (Fig. 1, scenario 2). In an attempt to maximize a conservative selection of potentially impactful compound heterozygous events, scenario 2 was not considered as an SNV-deletion event in the current study. Within these regions, we used the biomaRt package^38^ in R to identify genic regions for our downstream analyses; the output contained ~50.5% intronic sequence, and 16.5% sequence up and downstream from the outer exons, as well as the 3′ and 5′ UTRs. All genotyping results of variants within the deletion region were haploid, i.e., showing as homozygous calls. We excluded variants showing identical (“homozygous”) calls in both proband and deletion-transmitting parent (*n* = 276) under the assumption that parents were not affected with ASD. In order to identify homozygote reference alleles and missing genotypes, we used FixVcfMissingGenotypes^39^. We thus excluded variants that were not called (*n* = 76), based on the depth of coverage from the BAM files. Hence, after merging the VCFs files, we coded both homozygotes reference and genotypes not called as missing. After these quality control steps, we retained 109 SNVs identified in inherited deleted gene sequences.

### Statistical analyses

We designed our study to detect an overall difference in rates of compound heterozygous events between probands and transmitting parents among 47 complete trios and 102 proband–parent pairs. Hence, we combined all deleted gene sequence in probands and tallied the number of SNVs in this pooled proband sequence. Similarly, we calculated the rate of variants in the pooled deleted gene sequence of their deletion-transmitting parents. By design, the combined proband sequence is equal in identity and length as the combined transmitting parent sequence (see Fig. 2). Therefore, to test the difference between the number of variants in the proband and the transmitting parent sequences, we have used the chi-square test.

Further, we annotated the identified sequence variants using CADD scores^29^, a publicly available online tool that integrates multiple variables to calculate an estimation of the predicted deleteriousness of sequence variants in the human genome. The output metric of CADD is a scaled “PHRED” score, which relies on the ranking of the predicted deleteriousness in the context of all ~8.6 billion sequence variants in the human genome^29^. In the group of individuals in whom SNV-deletion events were identified, we used a burden test^40^ to compare the cumulative scaled CADD scores between probands and parents. More specifically, all the SNVs’ CADD scores (in inherited CNV deletion regions, Supplementary Table 1) were aggregated for each individual. In other words, we calculated the sum score of CADD scores of the SNVs in the regions of interest for each individual. We then used logistic regression to compare the aggregated CADD scores between probands and parents.

Subsequently, we combined two filters to select for variants that are putatively most deleterious: (1) a CADD-10 score (defined as SNVs at the 10th% of CADD scores) to select only those sequence variants predicted to be most deleterious;^29^ and (2) variants predicted to change the properties of the encoded protein (in our data: missense variants and or splice-site altering variants)^11,41,42^. We retained variants that were identified by either one or both of these two filters.

Because of these three analyses conducted (1) the difference between the number of variants in the proband and the transmitting parent sequences; (2) burden test; and (3) analysis of most deleterious SNVs, we considered *p* values < 0.05/3 (Bonferroni correction for multiple testing) as statistically significant.

The data analyzed for the current study is derived from the AGP^20^, available through dbGap (https://www.ncbi.nlm.nih.gov/projects/gap/cgi-bin/study.cgi?study_id=phs000267.v5.p2).

## Results

We obtained sequence data from 201 brain-relevant genes in 149 families (see Supplementary Table 2). For each family we restricted our analyses to the genes affected by the deletion transmitted in that family. We observed an average of 3.08 brain-relevant genes affected by a deletion per family. We identified a total of 109 SNVs in these deletions. There were 20 probands (13.4%) with at least one SNV-deletion compared with 16 deletion-transmitting parents (8.1%). There was a significant difference in distribution between probands and parents: 68 variants were identified in the pooled sequence of probands versus 41 variants in the pooled deletion-transmitting parent sequence (*X*^2^ = 6.69, *p* = 0.0097). Table 1 provides an overview of the identified SNVs in inherited deletion regions, along with their annotations. Supplementary Tables 1 and 3, and Supplementary Fig. 1 provide more detailed information, including distribution of variants and boundaries of the deletions involved in the observed SNV-deletion events. Of note, six probands in the subset of 47 complete trios carried a compound heterozygous event, which consisted of an inherited deletion and a de novo SNV (see Supplementary Table 4).

**Table 1:** Annotation of sequence variants (annotation by SnpEff).

	Sequence variants in probands	Sequence variants in deletion-transmitting parents
Type of sequence variant		
3′ UTR	3	1
Downstream gene	4	3
Intron	36	19
Missense	8	7
Missense variant and splice region variant	1	0
Non-coding exon variant	2	2
Splice region and Intron	2	0
Synonymous	7	7
Upstream gene	5	2
Total	68	41

3′ UTR: UTR variant of the 3′ UTR; Downstream gene: variant located at the 3′ boundary of a gene; Intron: variant occurring within an intron; Missense: variant that changes one or more bases, resulting in a different amino acid sequence but where the length is preserved; Non-coding exon: a sequence variant that changes non-coding exon sequence; Splice region: sequence variant in which a change has occurred within the region of the splice site, either within 1–3 bases of the exon or 3–8 bases of the intron; Synonymous: sequence variant where there is no resulting change to the encoded amino acid; Upstream gene: sequence variant located at the 5′ end of a gene. Splice region variants (all probands): rs1800340: chr16: 89771670; A > G, rs10253598: chr7: 92083703; A > T, rs1059830: chr1:1719358; A > G.

The burden test showed a significantly higher cumulative CADD score conferred by 68 SNVs observed in inherited deletions in 20 probands compared with 41 SNV-deletion events observed in 16 transmitting parents (*β* = 0.13, *p* = 1.0 × 10^−^^5^). However, the burden test applied to the entire sample, i.e., including the 129 probands and 180 parents without SNV-deletion events, was not significant (*β* = 0.019, *p* = 0.25).

Then we examined the SNVs yielded from the union of the two deleteriousness filters (Table 2). Of these 29 putatively most deleterious SNVs, 21 were detected in proband sequences versus 8 in parents (*X*^2^ = 5.82, *p* = 0.016; Supplementary Table 5). Post hoc we reiterated this analysis after omitting rs75355616 as this variant is located in a segmental duplication region overlapping with *PRAMEF4*, which implies highly homologous sequences elsewhere in the genome^43^, yielding unaltered results (20 SNVs in probands versus 8 in parents; *X*^2^ = 5.14, *p* = 0.023).

**Table 2:** Distribution of SNVs, after application of two filters on the total of 109 SNVs identified: (1) top 10% predicted most deleterious and, (2) missense or slice-site altering variants only.

		CADD-10 SNVs Top 10% predicted deleterious		Missense or splice-site altering SNVs		Top 10% and/or missense/splice altering SNVs		
Gene name	Chr: start–end (hg19)	Parents	Proband	Parents	Proband	Parents	Proband	Associated with phenotypes
ABCC6	16: 16242785–16317379	1	0	2	0	2	0	Pseudoxanthoma elasticum; Arterial calcification of infancy^48,49^
AF001548.6	16: 1582031–15826850	1	0	0	0	1	0	NA
AKAP9	7: 91570181–91739987	0	0	0	1	0	1	Long QT Syndrome 11^50,51^
CAMK2B	7: 44256749–44374176	0	1	0	0	0	1	Mental retardation, autosomal dominant, Phencyclidine abuse^52,53^
CDK11A	1: 1634169–1655766	0	1	0	1	0	1	Childhood endodermal sinus tumor, Neuroblastoma^54^
CTD-2245F17.6	19: 53743927–53745165	0	2	0	0	0	2	NA
FANCA	16: 89803957–89883065	0	1	0	1	0	2	Fanconi anemia, Neuroblastoma^55,56^
FKBP15	9: 115923286–115983641	1	0	1	0	1	0	NA
MYH11	16: 15797029–15950890	1	0	1	0	1	0	Aortic aneurysm, Familial thoracic aneurysm^57–59^
NDE1	16: 15737124–15820210	0	2	0	0	0	2	Microhydranencephaly, Lissencephaly, Hydranencephaly, Microlissencephaly^60,61^
OR2L1P	1: 248201474–248202607	0	1	0	0	0	1	NA
OR2L2	10: 3179920–3215003	0	1	2	0	2	0	NA
PITRM1-AS1	10: 3183793–3210164	0	0	0	0	0	1	NA
PPL	16: 4932508–5010742	1	0	1	0	1	0	Paraneoplastic pemphigus, Pemphigus foliaceus^62,63^
PRAMEF4	1: 12939033–12946025	0	0	0	1	0	1	NA
RP11-15A1.2	19: 43902001–43926545	0	2	0	0	0	2	NA
ZNF257	19: 22235254–22274282	0	1	0	1	0	1	NA
ZNF45	19: 44416781–44439430	0	0	0	2	0	2	NA
ZNF92	7: 64838712–64866038	0	0	0	4	0	4	NA
Total	5	12	7	11	8	21		

The third column aggregates the union of SNVs resulting from either filter (and/or).

## Discussion

This study provides tentative evidence for the role of a specific type of compound heterozygosity in the genetic architecture of ASD. Results indicate that in individuals with ASD, inherited deletions may co-occur more often with a predicted functional SNV affecting the remaining allele at the same locus than in their unaffected parents. Our burden analysis shows that, cumulatively, the burden of predicted deleteriousness inferred by variants on the remaining allele is significantly higher in probands than in their deletion-transmitting parents, providing further evidence for our “compound heterozygosity” hypothesis in ASD.

The pathogenic potential of some CNVs, in particular deletions, may sometimes be contingent on the presence of an additional genetic variant on the remaining allele. Vice versa, the phenotypic impact of the latter may in turn only be revealed when not compensated by a second wild-type allele, such as is the case in the presence of a deletion. A deletion, in such situation, can be said to “unmask the functional effect of a variant”^44^ which would otherwise have remained without phenotypic consequences. The compound nature implies a mutual rapport: a functional variant can equally be said to “uncover the pathogenicity of a deletion”. In the clinic, putatively pathogenic deletions identified in some patients often turn out to be inherited from seemingly unaffected parents^45^. This scenario strongly suggests the requirement of additional factors to mediate the pathogenic potential of the CNV. Although not currently applicable to clinical settings, we propose that the compound heterozygosity described in the current study is one of several mechanisms explaining variable penetrance of CNVs with known pathogenicity for ASD^34^.

Findings reported here are limited by the relatively small sample size. Given this, we restricted the statistical analysis in this work to only test the main hypothesis—that compound heterozygosity of a deletion and a functional sequence variant at the remaining allele occurs more often in patients with ASDs compared with the parents carrying the same deletion. In this study, we focused on deletions assuming a model of loss-of-function. This is a limitation by design, as duplications may also contribute to the etiology of ASD through dosage and gain-of-function. Arguably, compound heterozygous events may also occur under these scenarios. The annotation of SNVs included synonymous variants. In light of the overall small number of variants, we chose to retain this subset of SNVs in our analyses, even though they do not alter protein sequence and therefore have a lower probability of functional impact. In support of our approach, several recent studies suggest that synonymous variants can be pathogenic^46^. However, our main finding remained significant when comparing the burden of SNVs after excluding the synonymous variants (*X*^2^ = 7.67, *p* = 0.006). In addition, when we restricted the analyses to a subset of 29 variants predicted to be amongst the most deleterious variants in the genome (Supplementary Table 5), we observed a significantly higher burden of these in compound heterozygous events in probands compared with their unaffected parents. However, given our overall low event rate, we were not able to apply both filters (i.e., the intersection of CADD-10 and missense/splice-site altering variants) in a single analysis, which would have been a more stringent approach. The low overall event rate also prevents us from discriminating individual true versus false positive signals within the higher burden observed in probands. Given the limitations described above, we present our results as exploratory, to show the potential contribution of compound heterozygous events involving deletions. Hence, replication of our findings in independent studies is required: whole genome or exome sequencing would be the most appropriate method for such an endeavor^47^ within a sample with reliable matched CNV calls.

In conclusion, our results provide initial evidence for a role of compound heterozygosity in ASD. We propose that the compound heterozygosity described in the current study is one of several mechanisms explaining variable penetrance of CNVs, in particular deletions, with known pathogenicity for ASD. This mechanism can be taken into account in studies aiming to identify genetic variants contributing to ASD. Compound heterozygosity may be one factor that explains the frequently observed inconsistent phenotypic expression amongst carriers of the same putatively pathogenic deletion.

## Supplementary information

Supplementary Methods

Supplementary Figure and tables

## Data Availability

The data analyzed for the current study are derived from the Autism Genome Project, available through dbGap (https://www.ncbi.nlm.nih.gov/projects/gap/cgibin/study.cgi?study_id=phs000267.v5.p2). The data generated during the current study are not publicly available due to individual privacy concerns but are available from the corresponding author on reasonable request.
