# Supplementary material for: The role of rare compound heterozygous events in autism spectrum disorder
Source: Transl Psychiatry. 2020 Jun 22;10:204. doi: 10.1038/s41398-020-00866-7 (PMC7308334; doi:10.1038/s41398-020-00866-7)
Supplement: Supplementary file 1 — Supplementary Methods [file 41398_2020_866_MOESM1_ESM.docx]

**Supplementary Methods**

**Annotation of brain-relevance of genes**

We define “brain relevant genes” as those that show evidence of “brain relevance” from three sources: 1) gene ontology database, 2) expressed sequence tags (EST) annotation and 3) gene expression micro-arrays.

All gene co-ordinates were downloaded from the University of California Santa Cruz (UCSC) Reference Sequence (RefSeq; https://genome.ucsc.edu/cgi-bin/hgGateway). We limited to analysis to the super-gene; specifically, to reduce inflation for genes presenting two or more overlapping transcripts, we only selected the super-gene overlapping all others. The original RefSeq database consists of more than 30,000 records, however, after filtering for super-gene overlapping all others, the number of genes deflates to NrefSeq = 21,633.

We used the abovementioned three sources of information for our annotation of “brain-relevance”: The Venn diagram below describes the joint distribution of those three annotations. Using the concordance of these three data sources, we defined 6371 genes (29.5% of the total reference genes) as brain-relevant genes.

**1. Gene Ontology** (GO; http://geneontology.org)(1) provides an ontology of defined terms representing gene product properties. GOs classify in three groups, namely (1) the cellular component (parts of a cell or of its extracellular environment), (2) the molecular function (elemental activities of a gene product at the molecular level like binding, catalysis) and (3) the biological process (operations or sets of molecular events with a defined beginning and end, pertinent to the functioning of integrated living units like cells, tissues, organs, and organisms). With text mining, we selected GOs from the GO Consortium’s annotation and ontology toolkit(2) (AmiGO) database that match a number of brain-related text tokens (see Table 1 below).

**2. Expressed Sequence Tag (EST) Annotation.** An EST is a unique DNA sequence derived from a complementary DNA library, i.e. from a sequence which has been transcribed in some tissue or at some stage of development. The EST can be mapped, by a combination of genetic mapping procedures, to a unique locus in the genome and serves to identify that gene locus. As plain English describes the tissue provenance of every EST, here again we use text search tokens to annotate ESTs as brain-relevant or not. When mapped back to their genomic locations and matched against the RefSeq and CNV databases, this EST annotation enables to count the number of EST calls per gene transcript and per CNV. As the likelihood to call EST increases with the length of the transcripts or CNVs, we normalize by the genomic length the number of EST calls.

**3. Gene Expression.** We used a combination of Affymetrix gene expression analysis data to determine the brain relevance of genes (3). In short, this dataset was based on normalized expression data derived from ~37.5k samples from a range of human tissues (Affymetrix HG-U133 Plus 2.0). Text mining using Medical Subject Headings (MeSH) ontology was used to identify a subset of ~1.1k brain tissue samples. Expression levels were compared between this subset of brain tissue samples and the remaining ~36.4k samples; subsequently for each probe an area under the curve score was generated, with 0.5 equal expression in brain and non-brain tissue. Scores closer to one suggest highly brain-relevant probes, whereas scores near zero indicate brain-unrelated probes.

**Venn diagram**

Joint distribution of genes (N=21,633) with respect to gene expression (GE), gene ontology (GO) and brain ESTs (EST). At total of 20,303 genes were annotated by at least one source, whereas 1,330 genes were not annotated by either one of these three sources.


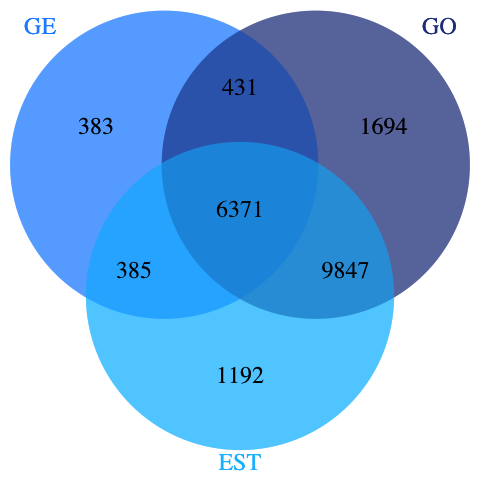


**Supplementary Methods Table 1.**

List of GO-terms used to annotate probably brain-relevance using the AmiGo database—a star indicates a match for any suffix (<http://amigo.geneontology.org>)

amygdala

brain*

callosum

caudate

cerebel*

cerebr*

cingulate

cns

coeruleus

cochlea

cortex

cortic*

csf

dendrit*

dura

forebrain

ganglia

ganglion

gyrus

head

hemispher

hippocamp*

hypothal*

mamillary

medulla*

meninges

mesencepholon

midbrain

nerv*

neur*

nigra

nucleus

oblongata

occipital

olfactory

pineal

pituit*

pons

putamen

spinal

striatum

subthalamic

telencephalon

thalam*

myelin

neurotransmitter

dopamin*

serotonin*

acetylcholin*

glutamat*

muscarin*

synap*

**References**

1. Ashburner M, Ball CA, Blake JA, Botstein D, Butler H, Cherry JM, et al. Gene ontology: tool for the unification of biology. The Gene Ontology Consortium. Nature genetics. 2000;25(1):25-9.

2. Carbon S, Ireland A, Mungall CJ, Shu S, Marshall B, Lewis S, et al. AmiGO: online access to ontology and annotation data. Bioinformatics. 2009;25(2):288-9.

3. Franke L, van Bakel H, Fokkens L, de Jong ED, Egmont-Petersen M, Wijmenga C. Reconstruction of a functional human gene network, with an application for prioritizing positional candidate genes. Am J Hum Genet. 2006;78(6):1011-25.
