# Supplementary material for: The role of rare compound heterozygous events in autism spectrum disorder
Source: Transl Psychiatry. 2020 Jun 22;10:204. doi: 10.1038/s41398-020-00866-7 (PMC7308334; doi:10.1038/s41398-020-00866-7)
Supplement: Supplementary file 2 — Supplementary Figure and tables [file 41398_2020_866_MOESM2_ESM.docx]

**Supplement to:**

**The role of rare compound heterozygous events in autism spectrum disorder**

**Lin et al.**

**Supplementary Table 1:** 109 SNV-deletions with deletion boundaries and gene affected by the deletion.

| **Fam ID** | **Carrier** | **chr** | **CNV start** | **CNV end** | **Gene Name** | **SNV**  **rsnumber** | **MAF^*^** | **PHRED** | **Annotation** |
| --- | --- | --- | --- | --- | --- | --- | --- | --- | --- |
| 3495 | parents | 1 | 246221677 | 246287577 | OR2L2 | rs112747916 | 0.051 | 0.551 | 3 prime UTR variant |
| 3495 | parents | 1 | 246221677 | 246287577 | OR2L2 | rs113312394 | 0.015 | 0.001 | missense variant |
| 3495 | parents | 1 | 246221677 | 246287577 | OR2L13 | rs180966267 | 0.002 | 4.45 | non coding exon variant |
| 13022 | parents | 1 | 12.812.943 | 13.193.467 | PRAMEF4 | rs201240342 | 0 | 0.455 | synonymous variant |
| 3495 | parents | 1 | 246221677 | 246287577 | OR2L2 | rs6658141 | 0.342 | 0.182 | missense variant |
| 3495 | parents | 1 | 246221677 | 246287577 | OR2L13 | rs72475095 | 0.114 | 3.005 | non coding exon variant |
| 3495 | parents | 1 | 246221677 | 246287577 | OR2L2 | rs73141398 | 0.051 | 0.11 | intron variant |
| 17017 | parents | 1 | 1610720 | 1653984 | CDK11A | rs874516 | 0.46 | 1.96 | downstream gene variant |
| 3484 | parents | 1 | 1610720 | 1656198 | CDK11A | rs874516 | 0.46 | 1.96 | downstream gene variant |
| 14134 | parents | 4 | 5645102 | 5680199 | EVC2 | rs751842 | 0.403 | 3.412 | intron variant |
| 3393 | parents | 6 | 152341582 | 152350825 | SYNE1 | rs2813561 | 0.206 | 4.406 | intron variant |
| 3393 | parents | 6 | 152341582 | 152350825 | SYNE1 | rs2813562 | 0.206 | 2.086 | intron variant |
| 3484 | parents | 8 | 18896754 | 18908145 | PSD3 | rs1386692 | 0.428 | 2.025 | intron variant |
| 3171 | parents | 9 | 114901022 | 115014100 | FKBP15 | rs10118429 | 0.281 | 7.508 | intron variant |
| 3171 | parents | 9 | 114901022 | 115014100 | FKBP15 | rs10435864 | 0.47 | 0.352 | synonymous variant |
| 3171 | parents | 9 | 114901022 | 115014100 | FKBP15 | rs10817454 | 0.299 | 5.281 | intron variant |
| 3171 | parents | 9 | 114901022 | 115014100 | FKBP15 | rs1133618 | 0.184 | 14.62 | missense variant |
| 3171 | parents | 9 | 114901022 | 115014100 | FKBP15 | rs12684417 | 0.287 | 3.236 | intron variant |
| 3171 | parents | 9 | 114901022 | 115014100 | FKBP15 | rs3810910 | 0.289 | 1.807 | synonymous variant |
| 3171 | parents | 9 | 114901022 | 115014100 | FKBP15 | rs4548256 | 0.186 | 2.291 | intron variant |
| 3171 | parents | 9 | 114901022 | 115014100 | FKBP15 | rs7865125 | 0.27 | 6.992 | intron variant |
| 3171 | parents | 9 | 114901022 | 115014100 | FKBP15 | rs7866432 | 0.2 | 0.194 | intron variant |
| 14358 | parents | 15 | 20301669 | 20839620 | CYFIP1 | rs2289819 | 0.201 | 5.269 | intron variant |
| 14358 | parents | 15 | 20301669 | 20839620 | CYFIP1 | rs2305093 | 0.19 | 6.173 | intron variant |
| 14370 | parents | 16 | 4888937 | 5140516 | PPL | rs1049205 | 0.447 | 23.1 | missense variant |
| 14412 | parents | 16 | 14687715 | 16270740 | MYH11 | rs1050113 | 0.304 | 14.37 | downstream gene variant |
| 14412 | parents | 16 | 14687715 | 16270740 | MYH11 | rs16967494 | 0.237 | 22.2 | missense variant |
| 14412 | parents | 16 | 14687715 | 16270740 | ABCC1 | rs2230671 | 0.188 | 6.703 | synonymous variant |
| 14412 | parents | 16 | 14687715 | 16270740 | ABCC6 | rs2238472 | 0.195 | 23 | missense variant |
| 14412 | parents | 16 | 14687715 | 16270740 | MYH11 | rs2272554 | 0.424 | 3.602 | synonymous variant |
| 14412 | parents | 16 | 14687715 | 16270740 | MYH11 | rs2280764 | 0.449 | 3.151 | intron variant |
| 3202 | parents | 16 | 16154972 | 16168752 | ABCC6 | rs2283508 | 0.377 | 6.604 | intron variant |
| 14335 | parents | 16 | 21856623 | 22337957 | POLR3E | rs2926362 | 0.38 | 3.835 | intron variant |
| 3202 | parents | 16 | 16154972 | 16168752 | ABCC6 | rs58760581 | 0.006 | 2.289 | intron variant |
| 14412 | parents | 16 | 14687715 | 16270740 | ABCC6 | rs8058694 | 0.358 | 0.003 | missense variant |
| 14256 | parents | 18 | 35213368 | 35299318 | RPL7AP66 | rs4800092 | 0.48 | 4.365 | upstream gene variant |
| 14312 | parents | 18 | 7678095 | 7698930 | PTPRM | rs8082894 | 0.234 | 5.175 | intron variant |
| 3017 | parents | 19 | 47990196 | 48435900 | PSG4 | rs3859474 | 0.367 | 0.5 | synonymous variant |
| 3017 | parents | 19 | 47990196 | 48435900 | PSG4 | rs3859476 | 0.413 | 1.113 | intron variant |
| 14260 | parents | 19 | 22041976 | 22144696 | AC003973.5 | rs387343 | 0.441 | 3.978 | upstream gene variant |
| 3017 | parents | 19 | 47990196 | 48435900 | PSG4 | rs9789333 | 0.402 | 9.072 | synonymous variant |
| 13003 | proband | 1 | 1610720 | 1653984 | CDK11A | rs1059830 | 0.5 | 23.4 | missense & splice region variant |
| 3495 | proband | 1 | 246221677 | 246287577 | OR2L13 | rs185245688 | 0.002 | 14.72 | non coding exon variant |
| 3495 | proband | 1 | 246221677 | 246287577 | OR2L2 | rs72763251 | 0.003 | 0.005 | intron variant |
| 13003 | proband | 1 | 1610720 | 1653984 | CDK11A | rs74223875 | 0 | 0.866 | intron variant |
| 13022 | proband | 1 | 12.812.943 | 13.193.467 | PRAMEF4 | rs75355616 | 0.216 | 0.002 | missense variant |
| 3478 | proband | 7 | 64305060 | 64758495 | ZNF92 | rs10239197 | 0.32 | 6.73 | missense variant |
| 3456 | proband | 7 | 64305060 | 64758495 | ZNF92 | rs10239197 | 0.32 | 6.73 | missense variant |
| 17025 | proband | 7 | 91429639 | 91458032 | AKAP9 | rs10253598 | 0.322 | 0.178 | splice region & intron variant |
| 3478 | proband | 7 | 64305060 | 64758495 | ZNF92 | rs10265083 | 0.309 | 4.155 | missense variant |
| 3456 | proband | 7 | 64305060 | 64758495 | ZNF92 | rs10265083 | 0.309 | 4.155 | missense variant |
| 13037 | proband | 7 | 44.245.623 | 44.280.233 | CAMK2B | rs1065359 | 0.342 | 11.46 | synonymous variant |
| 3478 | proband | 7 | 64305060 | 64758495 | ZNF92 | rs11979359 | 0.319 | 4.193 | intron variant |
| 3456 | proband | 7 | 64305060 | 64758495 | ZNF92 | rs4122883 | 0.311 | 3.248 | 3 prime UTR variant |
| 3478 | proband | 7 | 64305060 | 64758495 | ZNF92 | rs4122883 | 0.311 | 3.248 | 3 prime UTR variant |
| 3484 | proband | 8 | 18896754 | 18908145 | PSD3 | rs2632847 | 0.133 | 0.452 | intron variant |
| 3484 | proband | 8 | 18896754 | 18908145 | PSD3 | rs7003060 | 0.332 | 0.018 | synonymous variant |
| 3171 | proband | 9 | 114901022 | 115014100 | FKBP15 | rs3810909 | 0.2 | 0.877 | synonymous variant |
| 3171 | proband | 9 | 114901022 | 115014100 | FKBP15 | rs3827673 | 0.464 | 5.718 | intron variant |
| 3263 | proband | 10 | 3167320 | 3226016 | PITRM1 | rs3740607 | 0.426 | 11.12 | non coding exon variant |
| 3263 | proband | 10 | 3167320 | 3226016 | PITRM1 | rs7094698 | 0.284 | 1.342 | upstream gene variant |
| 3457 | proband | 13 | 49229284 | 49245892 | KPNA3 | rs12877931 | 0.2 | 8.438 | intron variant |
| 3457 | proband | 13 | 49229284 | 49245892 | KPNA3 | rs200661623 | 0.008 | 1.618 | intron variant |
| 17026 | proband | 15 | 29143717 | 29172089 | TRPM1 | rs2241493 | 0.168 | 0.178 | synonymous variant |
| 14412 | proband | 16 | 14687715 | 16270740 | MYH11 | rs1050162 | 0.374 | 14.25 | intron variant |
| 14412 | proband | 16 | 14687715 | 16270740 | MYH11 | rs1050163 | 0.373 | 11.23 | intron variant |
| 14412 | proband | 16 | 14687715 | 16270740 | MYH11 | rs11644832 | 0.221 | 0.745 | intron variant |
| 14412 | proband | 16 | 14687715 | 16270740 | MYH11 | rs11645883 | 0.221 | 2.829 | intron variant |
| 14382 | proband | 16 | 87792596 | 87839900 | ZNF778 | rs12926996 | 0.167 | 0.708 | downstream gene variant |
| 14382 | proband | 16 | 87792596 | 87839900 | ZNF778 | rs12926997 | 0.167 | 4.604 | downstream gene variant |
| 14412 | proband | 16 | 14687715 | 16270740 | ABCC1 | rs16967755 | 0.287 | 1.243 | intron variant |
| 14412 | proband | 16 | 14687715 | 16270740 | ABCC1 | rs16967758 | 0.284 | 1.653 | intron variant |
| 14246 | proband | 16 | 88288153 | 88453057 | ZNF276 | rs17233826 | 0.341 | 0.109 | 3 prime UTR variant |
| 14412 | proband | 16 | 14687715 | 16270740 | MYH11 | rs17284411 | 0.491 | 0.492 | intron variant |
| 14246 | proband | 16 | 88288153 | 88453057 | FANCA | rs1800285 | 0.348 | 3.317 | intron variant |
| 14246 | proband | 16 | 88288153 | 88453057 | FANCA | rs1800286 | 0.194 | 10.75 | intron variant |
| 14246 | proband | 16 | 88288153 | 88453057 | FANCA | rs1800287 | 0.341 | 0.888 | intron variant |
| 14246 | proband | 16 | 88288153 | 88453057 | FANCA | rs1800330 | 0.463 | 0.427 | intron variant |
| 14246 | proband | 16 | 88288153 | 88453057 | FANCA | rs1800337 | 0.358 | 1.522 | intron variant |
| 14246 | proband | 16 | 88288153 | 88453057 | FANCA | rs1800340 | 0.477 | 1.423 | splice region & intron variant |
| 14246 | proband | 16 | 88288153 | 88453057 | FANCA | rs1800359 | 0.185 | 0.453 | downstream gene variant |
| 14412 | proband | 16 | 14687715 | 16270740 | MYH11 | rs2075511 | 0.378 | 0.91 | upstream gene variant |
| 14412 | proband | 16 | 14687715 | 16270740 | MYH11 | rs2075514 | 0.221 | 3.26 | intron variant |
| 14412 | proband | 16 | 14687715 | 16270740 | MYH11 | rs2272552 | 0.264 | 1.918 | intron variant |
| 14377 | proband | 16 | 1757598 | 1763025 | MAPK8IP3 | rs2294618 | 0.168 | 0.154 | synonymous variant |
| 14412 | proband | 16 | 14687715 | 16270740 | RRN3 | rs2941259 | 0.384 | 3.261 | intron variant |
| 14412 | proband | 16 | 14687715 | 16270740 | RRN3 | rs2966179 | 0.396 | 1.988 | intron variant |
| 14246 | proband | 16 | 88288153 | 88453057 | FANCA | rs3743860 | 0.436 | 2.511 | intron variant |
| 14412 | proband | 16 | 14687715 | 16270740 | RRN3 | rs4985147 | 0.482 | 1.876 | intron variant |
| 14246 | proband | 16 | 88288153 | 88453057 | FANCA | rs6500452 | 0.48 | 3.058 | intron variant |
| 14246 | proband | 16 | 88288153 | 88453057 | FANCA | rs7190823 | 0.343 | 0.003 | synonymous variant |
| 14246 | proband | 16 | 88288153 | 88453057 | FANCA | rs7195066 | 0.385 | 3.273 | synonymous variant |
| 14246 | proband | 16 | 88288153 | 88453057 | ZNF276 | rs7195906 | 0.333 | 1.769 | downstream gene variant |
| 14309 | proband | 19 | 58386035 | 58434946 | ZNF677 | rs12984188 | 0.476 | 15.5 | upstream gene variant |
| 14309 | proband | 19 | 58386035 | 58434946 | ZNF677 | rs12984473 | 0.476 | 11.35 | upstream gene variant |
| 14260 | proband | 19 | 22041976 | 22144696 | ZNF257 | rs140340371 | 0.007 | 11.85 | missense variant |
| 14309 | proband | 19 | 58386035 | 58434946 | ZNF677 | rs2965240 | 0.498 | 8.042 | intron variant |
| 14260 | proband | 19 | 22041976 | 22144696 | ZNF257 | rs3752163 | 0.358 | 2.036 | intron variant |
| 14198 | proband | 19 | 22041976 | 22144696 | ZNF257 | rs3752163 | 0.358 | 2.036 | intron variant |
| 14394 | proband | 19 | 49102686 | 49183047 | ZNF45 | rs388685 | 0.466 | 9.393 | missense variant |
| 14394 | proband | 19 | 49102686 | 49183047 | ZNF45 | rs388706 | 0.426 | 0.109 | intron variant |
| 14394 | proband | 19 | 49102686 | 49183047 | ZNF45 | rs399098 | 0.466 | 5.219 | missense variant |
| 14394 | proband | 19 | 49102686 | 49183047 | ZNF45 | rs406968 | 0.426 | 15.18 | intron variant |
| 14394 | proband | 19 | 49102686 | 49183047 | ZNF45 | rs407731 | 0.427 | 11.15 | intron variant |
| 14394 | proband | 19 | 49102686 | 49183047 | ZNF45 | rs417699 | 0.411 | 1.56 | intron variant |
| 14394 | proband | 19 | 49102686 | 49183047 | RP11-15A1.2 | rs423320 | 0.426 | 4.159 | intron variant |
| 14394 | proband | 19 | 49102686 | 49183047 | ZNF45 | rs423752 | 0.426 | 0.205 | intron variant |
| 14394 | proband | 19 | 49102686 | 49183047 | ZNF45 | rs425221 | 0.426 | 0.462 | intron variant |
| 14309 | proband | 19 | 58386035 | 58434946 | ZNF677 | rs8113436 | 0.477 | 1.346 | upstream gene variant |

^*^ MAF (minor allele frequency) as reported in the 1000 Genome sequencing data.

**Supplementary table 2.** List of genes selected for target sequencing.

| **chr** | **bp:start** | **bp:stop** | **gene** | **EnsembleID** |
| --- | --- | --- | --- | --- |
| 1 | 1570603 | 1590473 | *CDK11B* | ENSG00000248333 |
| 1 | 1592939 | 1624167 | *SLC35E2B* | ENSG00000189339 |
| 1 | 1631369 | 1633249 | *MMP23A* | ENSG00000215914 |
| 1 | 48998527 | 50489585 | *AGBL4* | ENSG00000186094 |
| 1 | 49193195 | 49242641 | *BEND5* | ENSG00000162373 |
| 1 | 10270863 | 10441661 | *KIF1B* | ENSG00000054523 |
| 1 | 1634169 | 1655766 | *CDK11A* | ENSG00000008128 |
| 1 | 169890467 | 170054349 | *KIFAP3* | ENSG00000075945 |
| 1 | 180244515 | 180472089 | *ACBD6* | ENSG00000135847 |
| 1 | 180407449 | 180407525 | *MIR3121* | ENSG00000265435 |
| 1 | 84543745 | 84704181 | *PRKACB* | ENSG00000142875 |
| 1 | 100315640 | 100389579 | *AGL* | ENSG00000162688 |
| 1 | 248100493 | 248264224 | *OR2L13* | ENSG00000196071 |
| 1 | 248112160 | 248113098 | *OR2L8* | ENSG00000196936 |
| 1 | 248128535 | 248129641 | *OR2AK2* | ENSG00000187080 |
| 1 | 248153569 | 248154506 | *OR2L1P* | ENSG00000224227 |
| 1 | 248201474 | 248202607 | *OR2L2* | ENSG00000203663 |
| 1 | 248223984 | 248224922 | *OR2L3* | ENSG00000198128 |
| 1 | 235294949 | 235491534 | *ARID4B* | ENSG00000054267 |
| 1 | 7844380 | 7905237 | *PER3* | ENSG00000049246 |
| 2 | 212240446 | 213403565 | *ERBB4* | ENSG00000178568 |
| 2 | 213290987 | 213291084 | *MIR548F2* | ENSG00000221782 |
| 2 | 214149113 | 215275225 | *SPAG16* | ENSG00000144451 |
| 2 | 241653181 | 241759725 | *KIF1A* | ENSG00000130294 |
| 3 | 151531825 | 151546276 | *AADAC* | ENSG00000114771 |
| 3 | 21459915 | 22414812 | *ZNF385D* | ENSG00000151789 |
| 3 | 2140497 | 3099645 | *CNTN4* | ENSG00000144619 |
| 3 | 1134260 | 1445901 | *CNTN6* | ENSG00000134115 |
| 3 | 114056941 | 114866118 | *ZBTB20* | ENSG00000181722 |
| 3 | 6811688 | 7783215 | *GRM7* | ENSG00000196277 |
| 4 | 5544499 | 5711275 | *EVC2* | ENSG00000173040 |
| 4 | 20730239 | 21950422 | *KCNIP4* | ENSG00000185774 |
| 4 | 158125334 | 158287227 | *GRIA2* | ENSG00000120251 |
| 4 | 71768043 | 71888166 | *MOBKL1A* | ENSG00000173542 |
| 4 | 93225550 | 94695707 | *GRID2* | ENSG00000152208 |
| 4 | 174189311 | 174189384 | *MIR548T* | ENSG00000221296 |
| 4 | 85590704 | 85887544 | *WDFY3* | ENSG00000163625 |
| 5 | 31639517 | 32111037 | *PDZD2* | ENSG00000133401 |
| 5 | 31936208 | 31936265 | *MIR4279* | ENSG00000266243 |
| 5 | 69711179 | 69743885 | *GTF2H2B* | ENSG00000226259 |
| 5 | 68856035 | 68890550 | *GTF2H2C* | ENSG00000183474 |
| 5 | 69477955 | 69510579 | *GTF2H2D* | ENSG00000262261 |
| 5 | 10971952 | 11904155 | *CTNND2* | ENSG00000169862 |
| 5 | 660883 | 693510 | *TPPP* | ENSG00000171368 |
| 5 | 9641417 | 9903938 | *LOC285692* | ENSG00000249781 |
| 5 | 70220768 | 70249769 | *SMN1* | ENSG00000172062 |
| 5 | 69345350 | 69374349 | *SMN2* | ENSG00000205571 |
| 5 | 54603588 | 54721409 | *SKIV2L2* | ENSG00000039123 |
| 5 | 64064757 | 64314590 | *CWC27* | ENSG00000153015 |
| 5 | 82373317 | 82649606 | *XRCC4* | ENSG00000152422 |
| 6 | 128029217 | 128239776 | *THEMIS* | ENSG00000172673 |
| 6 | 161768452 | 163148803 | *PARENTK2* | ENSG00000185345 |
| 6 | 291630 | 351355 | *DUSP22* | ENSG00000112679 |
| 6 | 34385231 | 34393902 | *RPS10* | ENSG00000124614 |
| 6 | 34247456 | 34360451 | *NUDT3* | ENSG00000272325 |
| 6 | 34256547 | 34393825 | *RPS10-NUDT3* | ENSG00000270800 |
| 6 | 151977826 | 152450754 | *ESR1* | ENSG00000091831 |
| 7 | 64838712 | 64866038 | *ZNF92* | ENSG00000146757 |
| 7 | 79998891 | 80308593 | *CD36* | ENSG00000135218 |
| 7 | 142880512 | 142881528 | *TAS2R39* | ENSG00000236398 |
| 7 | 74306894 | 74366314 | *PMS2P5* | ENSG00000123965 |
| 7 | 94536514 | 94925727 | *PPP1R9A* | ENSG00000158528 |
| 7 | 145813453 | 148118090 | *CNTNAP2* | ENSG00000174469 |
| 7 | 147075109 | 147075213 | *MIR548F4* | ENSG00000221442 |
| 7 | 91570181 | 91739987 | *AKAP9* | ENSG00000127914 |
| 7 | 127292234 | 127732661 | *SND1* | ENSG00000197157 |
| 7 | 127667124 | 127672160 | *LRRC4* | ENSG00000128594 |
| 7 | 127721913 | 127722012 | *MIR593* | ENSG00000207588 |
| 7 | 143955700 | 143956815 | *OR2A7* | ENSG00000243896 |
| 7 | 102178365 | 102213103 | *POLR2J3* | ENSG00000168255 |
| 7 | 102191679 | 102202755 | *SPDYE2* | ENSG00000205238 |
| 7 | 102290772 | 102301847 | *SPDYE2L* | ENSG00000173678 |
| 7 | 102277472 | 102283238 | *UPK3BL* | ENSG00000267368 |
| 7 | 102277496 | 102312088 | *POLR2J2* | ENSG00000267645 |
| 7 | 110303110 | 111202573 | *IMMP2L* | ENSG00000184903 |
| 7 | 110731062 | 110765510 | *LRRN3* | ENSG00000173114 |
| 8 | 31496902 | 32622548 | *NRG1* | ENSG00000157168 |
| 8 | 18384811 | 18942240 | *PSD3* | ENSG00000156011 |
| 8 | 7572965 | 7575975 | *FAM90A14* | ENSG00000189393 |
| 8 | 7137292 | 7140766 | *FAM90A13* | ENSG00000223885 |
| 8 | 7611204 | 7614214 | *FAM90A19* | ENSG00000237122 |
| 8 | 7580613 | 7591823 | *FAM90A18* | ENSG00000231656 |
| 8 | 7595909 | 7598918 | *FAM90A8* | ENSG00000224710 |
| 8 | 7618852 | 7621862 | *FAM90A9* | ENSG00000235825 |
| 8 | 7626499 | 7629510 | *FAM90A10* | ENSG00000254597 |
| 8 | 7213039 | 7243080 | *ZNF705G* | ENSG00000215372 |
| 8 | 19171128 | 19253729 | *SH2D4A* | ENSG00000104611 |
| 8 | 50822349 | 51706678 | *SNTG1* | ENSG00000147481 |
| 8 | 145159402 | 145162514 | *MAF1* | ENSG00000179632 |
| 8 | 98656407 | 98740998 | *MTDH* | ENSG00000147649 |
| 8 | 38127215 | 38239790 | *WHSC1L1* | ENSG00000147548 |
| 8 | 146198975 | 146228281 | *ZNF252* | ENSG00000196922 |
| 8 | 146220272 | 146220926 | *TMED10P1* | ENSG00000254618 |
| 9 | 125273081 | 125274022 | *OR1J2* | ENSG00000197233 |
| 9 | 125281420 | 125282361 | *OR1J4* | ENSG00000239590 |
| 9 | 135937365 | 135947248 | *CEL* | ENSG00000170835 |
| 9 | 5334969 | 5339873 | *RLN1* | ENSG00000107018 |
| 9 | 88161455 | 88356944 | *AGTPBP1* | ENSG00000135049 |
| 9 | 72042446 | 72287222 | *APBA1* | ENSG00000107282 |
| 9 | 115923286 | 115983641 | *FKBP15* | ENSG00000119321 |
| 9 | 2717502 | 2730037 | *KCNV2* | ENSG00000168263 |
| 9 | 3824127 | 4348392 | *GLIS3* | ENSG00000107249 |
| 9 | 3898642 | 3901248 | *C9orf70* | ENSG00000237009 |
| 10 | 55562531 | 57387702 | *PCDH15* | ENSG00000150275 |
| 10 | 1085848 | 1095110 | *IDI1* | ENSG00000067064 |
| 10 | 22604903 | 22609235 | *COMMD3* | ENSG00000148444 |
| 10 | 22605317 | 22618471 | *COMMD3-BMI1* | ENSG00000269897 |
| 10 | 22610140 | 22620413 | *BMI1* | ENSG00000168283 |
| 10 | 52750945 | 54058110 | *PRKG1* | ENSG00000185532 |
| 10 | 53059333 | 53059415 | *MIR605* | ENSG00000207813 |
| 10 | 53455247 | 53459355 | *CSTF2T* | ENSG00000177613 |
| 10 | 1228073 | 1779670 | *ADARB2* | ENSG00000185736 |
| 10 | 1568832 | 1599179 | *NCRNA00168* | ENSG00000205696 |
| 10 | 33466420 | 33625190 | *NRP1* | ENSG00000099250 |
| 10 | 135175984 | 135187193 | *ECHS1* | ENSG00000127884 |
| 10 | 135185060 | 135185167 | *MIR3944* | ENSG00000265395 |
| 10 | 135192695 | 135205198 | *PAOX* | ENSG00000148832 |
| 10 | 135207598 | 135234811 | *MTG1* | ENSG00000148824 |
| 10 | 3179920 | 3215003 | *PITRM1* | ENSG00000107959 |
| 10 | 22045466 | 22292698 | *DNAJC1* | ENSG00000136770 |
| 10 | 75910960 | 76469061 | *ADK* | ENSG00000156110 |
| 11 | 8959119 | 8964580 | *ASCL3* | ENSG00000176009 |
| 11 | 95709762 | 96076344 | *MAML2* | ENSG00000184384 |
| 11 | 96074602 | 96074690 | *MIR1260B* | ENSG00000266192 |
| 11 | 19372271 | 20143144 | *NAV2* | ENSG00000166833 |
| 11 | 19532437 | 19541443 | *FLJ13439* | ENSG00000254622 |
| 11 | 19732480 | 19734165 | *LOC100126784* | ENSG00000270607 |
| 12 | 124456392 | 124499986 | *ZNF664* | ENSG00000179195 |
| 12 | 124457788 | 124800570 | *ZNF664-FAM101A* | ENSG00000178882 |
| 12 | 27175479 | 27219276 | *MED21* | ENSG00000152944 |
| 13 | 72012098 | 72441330 | *DACH1* | ENSG00000165659 |
| 13 | 93879095 | 95059655 | *GPC6* | ENSG00000183098 |
| 13 | 53418109 | 53422775 | *PCDH8* | ENSG00000136099 |
| 13 | 50273447 | 50367057 | *KPNA3* | ENSG00000102753 |
| 13 | 45694650 | 45858237 | *GTF2F2* | ENSG00000188342 |
| 13 | 45766988 | 45775175 | *KCTD4* | ENSG00000180332 |
| 14 | 21756098 | 21819460 | *RPGRIP1* | ENSG00000092200 |
| 14 | 19983559 | 20020272 | *POTEM* | ENSG00000187537 |
| 14 | 20181104 | 20182079 | *OR11H2* | ENSG00000258453 |
| 14 | 20215587 | 20216528 | *OR4Q3* | ENSG00000182652 |
| 14 | 106383838 | 106391825 | *KIAA0125* | ENSG00000226777 |
| 14 | 66974125 | 67648520 | *GPHN* | ENSG00000171723 |
| 15 | 43891596 | 44010458 | *STRC* | ENSG00000242866 |
| 15 | 56379478 | 56535483 | *RFX7* | ENSG00000181827 |
| 15 | 22892005 | 23006016 | *CYFIP1* | ENSG00000068793 |
| 15 | 31293264 | 31453476 | *TRPM1* | ENSG00000134160 |
| 15 | 31357235 | 31357344 | *MIR211* | ENSG00000207702 |
| 16 | 78133310 | 79246564 | *WWOX* | ENSG00000186153 |
| 16 | 6069095 | 7763340 | *RBFOX1* | ENSG00000078328 |
| 16 | 89786808 | 89807311 | *ZNF276* | ENSG00000158805 |
| 16 | 89803957 | 89883065 | *FANCA* | ENSG00000187741 |
| 16 | 11400297 | 11400384 | *MIR548H2* | ENSG00000221801 |
| 16 | 2961938 | 3001209 | *FLYWCH1* | ENSG00000059122 |
| 16 | 22308730 | 22346424 | *POLR3E* | ENSG00000058600 |
| 16 | 22357257 | 22448486 | *CDR2* | ENSG00000140743 |
| 16 | 3661729 | 3730144 | *DNASE1* | ENSG00000213918 |
| 16 | 4932508 | 5010742 | *PPL* | ENSG00000118898 |
| 16 | 1756184 | 1820318 | *MAPK8IP3* | ENSG00000138834 |
| 16 | 1784986 | 1785067 | *MIR3177* | ENSG00000265820 |
| 16 | 89284118 | 89295363 | *ZNF778* | ENSG00000170100 |
| 16 | 15131710 | 15149921 | *NTAN1* | ENSG00000157045 |
| 16 | 15153879 | 15188174 | *RRN3* | ENSG00000085721 |
| 16 | 15797029 | 15950890 | *MYH11* | ENSG00000133392 |
| 16 | 16043434 | 16236931 | *ABCC1* | ENSG00000103222 |
| 16 | 16242785 | 16317379 | *ABCC6* | ENSG00000091262 |
| 16 | 32888790 | 32896822 | *SLC6A10P* | ENSG00000214617 |
| 16 | 32684852 | 32688053 | *TP53TG3* | ENSG00000183632 |
| 16 | 33204156 | 33264727 | *LOC653550* | ENSG00000205457 |
| 16 | 33261515 | 33264716 | *TP53TG3B* | ENSG00000261509 |
| 16 | 67596310 | 67673086 | *CTCF* | ENSG00000102974 |
| 17 | 77085427 | 77512230 | *RBFOX3* | ENSG00000267483 |
| 17 | 44370099 | 44415160 | *LRRC37A* | ENSG00000176681 |
| 17 | 19912657 | 20222339 | *SPECC1* | ENSG00000128487 |
| 17 | 15531274 | 15587625 | *TRIM16* | ENSG00000221926 |
| 17 | 65821640 | 65980494 | *BPTF* | ENSG00000262858 |
| 17 | 49039535 | 49198226 | *SPAG9* | ENSG00000008294 |
| 18 | 36786888 | 37380282 | *LOC647946* | ENSG00000267374 |
| 18 | 7566780 | 8406859 | *PTPRM* | ENSG00000173482 |
| 18 | 8360818 | 8367032 | *LOC100192426* | ENSG00000266149 |
| 19 | 22235254 | 22274282 | *ZNF257* | ENSG00000197134 |
| 19 | 53731577 | 53758151 | *ZNF677* | ENSG00000197928 |
| 19 | 57874845 | 57890933 | *ZNF547* | ENSG00000152433 |
| 19 | 57901218 | 57912786 | *ZNF548* | ENSG00000188785 |
| 19 | 44416781 | 44439430 | *ZNF45* | ENSG00000124459 |
| 19 | 44455375 | 44471861 | *ZNF221* | ENSG00000159905 |
| 19 | 44472014 | 44502477 | *ZNF155* | ENSG00000204920 |
| 19 | 43326015 | 43331030 | *LOC100289650* | ENSG00000225877 |
| 19 | 43343520 | 43359870 | *PSG10P* | ENSG00000248257 |
| 19 | 43568363 | 43587197 | *PSG2* | ENSG00000242221 |
| 19 | 43670408 | 43690688 | *PSG5* | ENSG00000204941 |
| 19 | 43696854 | 43711451 | *PSG4* | ENSG00000243137 |
| 19 | 43715943 | 43752798 | *LOC284344* | ENSG00000241104 |
| 20 | 35624752 | 35724398 | *RBL1* | ENSG00000080839 |
| 20 | 59827482 | 60515673 | *CDH4* | ENSG00000179242 |
| 20 | 32951041 | 33099198 | *ITCH* | ENSG00000078747 |
| 20 | 62189439 | 62205592 | *PRIC285* | ENSG00000130589 |
| 21 | 34001069 | 34100359 | *SYNJ1* | ENSG00000159082 |
| 22 | 30279144 | 30426855 | *MTMR3* | ENSG00000100330 |
| 22 | 40806285 | 41032706 | *MKL1* | ENSG00000196588 |
| 22 | 24299601 | 24303373 | *GSTT2B* | ENSG00000133433 |
| 22 | 24322339 | 24326106 | *GSTT2* | ENSG00000099984 |

**Supplementary Table 3:** Distribution of 109 SNV-deletion events in proband and CNV-transmitting parents. Genes occurring in more than one family are shown in bold.

| Family ID | Gene | N variants in probands | N variants in transmitting parents |
| --- | --- | --- | --- |
| 14412 | *ABCC1* | 2 | 1 |
| **14412** | ***ABCC6*** | **0** | **2** |
| **3202** | ***ABCC6*** | **0** | **2** |
| **14260** | ***AC003973.5*** | **0** | **1** |
| **14412** | ***AF001548.5*** | **1** | **0** |
| 14412 | *AF001548.6* | 0 | 1 |
| 17025 | *AKAP9* | 1 | 0 |
| 13037 | *CAMK2B* | 1 | 0 |
| **13003** | ***CDK11A*** | **2** | **0** |
| **14309** | ***CTD-2245F17.6*** | **3** | **0** |
| 14358 | *CYFIP1* | 0 | 2 |
| 14134 | *EVC2* | 0 | 1 |
| 14246 | *FANCA* | 11 | 0 |
| 3171 | *FKBP15* | 2 | 9 |
| 3457 | *KPNA3* | 2 | 0 |
| 14377 | *MAPK8IP3* | 1 | 0 |
| **17017** | ***MMP23A*** | **0** | **1** |
| **3484** | ***MMP23A*** | **0** | **1** |
| 14412 | *MYH11* | 5 | 3 |
| 14412 | *NDE1* | 2 | 0 |
| 3495 | *OR2L13* | 1 | 1 |
| 3495 | *OR2L1P* | 1 | 1 |
| 3495 | *OR2L2* | 0 | 3 |
| 3495 | *OR2L6P* | 0 | 1 |
| 3263 | *PITRM1-AS1* | 2 | 0 |
| 14335 | *POLR3E* | 0 | 1 |
| 14370 | *PPL* | 0 | 1 |
| 13022 | *PRAMEF4* | 1 | 1 |
| 3484 | *PSD3* | 2 | 1 |
| 3017 | *PSG4* | 0 | 3 |
| 14312 | *PTPRM* | 0 | 1 |
| 14394 | *RP11-15A1.2* | 7 | 0 |
| 14328 | *RP11-46C24.6* | 2 | 0 |
| 14256 | *RPL7AP66* | 0 | 1 |
| 14412 | *RRN3* | 3 | 0 |
| 3393 | *SYNE1* | 0 | 2 |
| 17026 | *TRPM1* | 1 | 0 |
| **14198** | ***ZNF257*** | **1** | **0** |
| **14260** | ***ZNF257*** | **2** | **0** |
| 14246 | *ZNF276* | 2 | 0 |
| 14394 | *ZNF45* | 2 | 0 |
| 14309 | *ZNF677* | 1 | 0 |
| **3456** | ***ZNF92*** | **3** | **0** |
| **3478** | ***ZNF92*** | **4** | **0** |
| **Total** |  | **68** | **41** |

**Supplementary table 4.** Out of a subset of 47 complete trios, in six probands the compound heterozygous event consisted of an inherited deletion and a *de novo* SNV.

| **SNV**  **rsnumber** | **FAMID** | **Chr** | **pos** | **Variant**  **allele**  **in proband** | **Gene**  **name** | **Genotype parent not transmitting the deletion** |
| --- | --- | --- | --- | --- | --- | --- |
| rs200661623 | 3457 | 13 | 50299649 | A | KPNA3 | C/C |
| rs2294618 | 14377 | 16 | 1814391 | C | MAPK8IP3 | T/T |
| rs1059830 | 13003 | 1 | 1650797 | G | CDK11A | A/A |
| rs2632847 | 3484 | 8 | 18659928 | G | PSD3 | A/A |
| rs7003060 | 3484 | 8 | 18729818 | G | PSD3 | T/T |

**Supplementary table 5.** The contingency table of 29 sequence variants annotated as missense / splice site altering variants and/or CADD-10. Genetic changes occurring in more than one family are shown in bold.

| **SNV**  **rsnumber** | **FAM**  **ID** | **Gene Name** | **Annotation** | **PHRED** | **Observed in** |
| --- | --- | --- | --- | --- | --- |
| rs2238472 | 14412 | *ABCC6* | missense variant | 23 | parents |
| rs113312394 | 3495 | *OR2L2* | missense variant | 0.001 | parents |
| rs8058694 | 14412 | *ABCC6* | missense variant | 0.003 | parents |
| rs6658141 | 3495 | *OR2L2* | missense variant | 0.182 | parents |
| rs1050113 | 14412 | *AF001548.6* | downstream gene variant | 14.37 | parents |
| rs1133618 | 3171 | *FKBP15* | missense variant | 14.62 | parents |
| rs16967494 | 14412 | *MYH11* | missense variant | 22.2 | parents |
| rs1049205 | 14370 | *PPL* | missense variant | 23.1 | parents |
| rs1800340 | 14246 | *FANCA* | splice region & intron variant | 1.423 | proband |
| **rs10265083** | 3478 | *ZNF92* | missense variant | 4.155 | proband |
| **rs10265083** | 3456 | *ZNF92* | missense variant | 4.155 | proband |
| rs399098 | 14394 | *ZNF45* | missense variant | 5.219 | proband |
| rs388685 | 14394 | *ZNF45* | missense variant | 9.393 | proband |
| rs75355616^1^ | 13022 | *PRAMEF4* | missense variant | 0.002 | proband |
| rs10253598 | 17025 | *AKAP9* | splice region & intron variant | 0.178 | proband |
| rs1800286 | 14246 | *FANCA* | intron variant | 10.75 | proband |
| rs3740607 | 3263 | *PITRM1-AS1* | non coding exon variant | 11.12 | proband |
| rs407731 | 14394 | *RP11-15A1.2* | intron variant | 11.15 | proband |
| rs1050163 | 14412 | *NDE1* | intron variant | 11.23 | proband |
| rs12984473 | 14309 | *CTD-2245F17.6* | upstream gene variant | 11.35 | proband |
| rs1065359 | 13037 | *CAMK2B* | synonymous variant | 11.46 | proband |
| rs140340371 | 14260 | *ZNF257* | missense variant | 11.85 | proband |
| rs1050162 | 14412 | *NDE1* | intron variant | 14.25 | proband |
| rs185245688 | 3495 | *OR2L1P* | non coding exon variant | 14.72 | proband |
| rs406968 | 14394 | *RP11-15A1.2* | intron variant | 15.18 | proband |
| rs12984188 | 14309 | *CTD-2245F17.6* | upstream gene variant | 15.5 | proband |
| rs1059830 | 13003 | *CDK11A* | missense & splice region variant | 23.4 | proband |
| **rs10239197** | 3478 | *ZNF92* | missense variant | 6.73 | proband |
| **rs10239197** | 3456 | *ZNF92* | missense variant | 6.73 | proband |


^1^ rs75355616 is located in a segmental duplication region overlapping with *PRAMEF4.*

**Supplementary Figure 1:** Minor Allele Frequency of 109 SNV-deletion events in proband and CNV-transmitting parents, as reported in the 1000 Genome sequencing data.
